# Transcriptomic and proteomic signatures underlying nymphal adaptation and foam production in the forage pest *M* *ahanarva spectabilis*

**DOI:** 10.1111/imb.70050

**Published:** 2026-06-12

**Authors:** Monique da Silva Bonjour, Angelo José Rinaldi, Eulálio Gutemberg Dias dos Santos, Gabriely Teixeira Bhering Faria, Ana Márcia Escocard de Azevedo Manhães, Jorge Fernando Pereira, Alexander Machado Auad, Maria Goreti de Almeida Oliveira, Humberto Josué de Oliveira Ramos

**Affiliations:** ^1^ Laboratory of Enzymology and Biochemistry of Proteins and Peptides, Department of Biochemistry and Molecular Biology Universidade Federal de Viçosa, UFV, BIOAGRO/INCT‐IPP Viçosa Brazil; ^2^ Embrapa Dairy Cattle Juiz de Fora Brazil; ^3^ Núcleo de Análise de Biomoléculas, NuBioMol Universidade Federal de Viçosa Viçosa Brazil

**Keywords:** detoxification enzymes, forage grasses, plant–insect interactions, proteomics, secretome, transcriptomics

## Abstract

The spittlebug *Mahanarva spectabilis* (Distant, 1909) (Hemiptera: Cercopidae) is an important pest of forage grasses in South America, where its nymphs cause pasture damage by feeding on xylem sap and producing a characteristic foam that protects them against environmental stressors. To investigate the molecular basis of this adaptation, we integrated RNA‐seq analysis of nymphs with LC–MS/MS proteomics of the Batelli gland, the primary source of foam secretion. De novo assembly of 100,666 unigenes revealed broad functional diversity, with strong representation of detoxification enzymes (CYP450s, GSTs, UGTs, carboxylesterases), transporters and ion pumps, cuticle proteins, and stress‐ and immunity‐related genes. Nearly 16% of loci exhibited alternative splicing, particularly within detoxification, chemosensory and osmoregulatory gene families, highlighting evidence of transcriptomic variability. Signal peptide and secreted protein predictions identified 168 high‐confidence candidate secreted proteins, including detoxification enzymes, proteases, structural proteins and immune‐related factors, several of which are consistent with antimicrobial and surfactant‐related functions. Proteomic profiling of the Batelli gland confirmed 500 proteins, enriched in chaperones, metabolic enzymes, detoxification pathways and osmoregulatory components, with the most abundant proteins corresponding to Hsp70 chaperones, ATP synthases, cuticle proteins and carbonic anhydrases. Together, these results provide an integrative transcriptomic and proteomic overview for *M. spectabilis* nymphs, highlighting genes and proteins associated with xylem feeding, foam production and responses potentially related to environmental stress tolerance. This comprehensive dataset not only advances the understanding of spittlebug biology but also identifies candidate molecular targets that may inform innovative strategies for controlling nymphal stages and mitigating spittlebug damage in forage systems.

## Graphical abstract

- RNA‐seq and Batelli gland proteomics of fifth‐instar *Mahanarva spectabilis* nymphs reveal transcripts and proteins associated with xylem feeding, foam production and environmental interaction.
- Functional annotation identified genes involved in osmoregulation, detoxification, chemosensation and stress responses, while proteomic analysis confirmed secreted and structural components of the foam‐producing gland.
- This integrative dataset provides a reference framework for molecular studies of spittlebug biology.

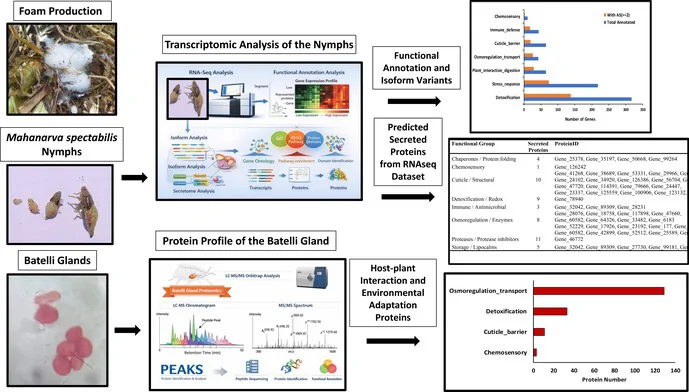

## INTRODUCTION

The spittlebug *Mahanarva spectabilis* (Distant, 1909) (Hemiptera: Cercopidae) is an insect pest of forage grasses, where livestock production depends largely on pasture‐based systems. Infestations of this insect reduce plant vigour and delay regrowth, leading to major yield and economic losses. Both nymphs and adults feed on xylem sap, but the nymphal stage is particularly harmful because of its feeding at the basal regions of plants. Effective control of the nymphal stage is critical, as it directly reduces the emergence of adults and the spread of infestations in pasture ecosystems (Resende et al., 2012).

A distinctive feature of spittlebug nymphs is the production of a frothy secretion that encases their bodies. This foam, produced mainly by the Batelli gland in combination with Malpighian tubule excretions, provides multiple protective functions (Farina et al., 2022; Marshall, 1965; Zhang et al., 2024). It acts as a physical and biochemical barrier against desiccation, temperature fluctuations, predators and microbial colonization, while creating a buffered microenvironment that supports nymphal survival under exposed field conditions (Auad et al., 2012; Alzani et al., 2023; Hoch et al., 2024; Sahayaraj et al., 2021; Tonelli et al., 2018; Tonelli et al., 2020). Studies on related species, such as *Mahanarva fimbriolata* and *Poophilus costalis*, have shown that spittlebug foams contain proteins, carbohydrates and lipids with insulating and antimicrobial properties (Sahayaraj et al., 2021; Tonelli et al., 2018). Nevertheless, the molecular mechanisms underlying foam production and its functional roles remain poorly characterized.

Advances in high‐throughput RNA sequencing (RNA‐seq) and proteomics now allow comprehensive functional characterization of insect transcriptomes and secretomes. Transcriptomic studies in hemipterans, such as *Empoasca vitis* (Shao et al., 2017), *Myzus persicae* (Sharma et al., 2024) and *Lipaphis erysimi* (Sharma et al., 2024), have revealed expansions in detoxification, osmoregulatory and chemosensory gene families, underscoring their role in plant adaptation. Proteomic approaches combined with transcriptomics have also been applied to salivary secretions in aphids (*Pseudoregma bambucicola*) (Zhang et al., 2023), ticks (*Ornithodoros erraticus*) (Pérez‐sánchez et al., 2022) and even specialized structures in leafhoppers (*Nephotettix cincticeps*) (Wu et al., 2023), identifying enzymes, effectors and structural proteins crucial for insect–plant interactions. These studies collectively highlight how integrative omics approaches can uncover the molecular strategies that insects employ to adapt to their ecological niches.

Despite the agronomic relevance of *M. spectabilis*, few molecular studies have targeted its nymphal stage or the Batelli gland, which is responsible for producing the protective foam. Here, we combined RNA‐seq analysis of *M. spectabilis* nymphs with LC–MS/MS proteomic profiling of the Batelli gland to provide the first integrated molecular characterization of foam production in spittlebugs. Genes and proteins were annotated and categorized into functional classes including detoxification, stress response, chemosensation, osmoregulation, immunity and cuticle remodelling. By linking transcriptomic signatures with proteomic evidence of secreted proteins, this study not only enhances our understanding of spittlebug biology but also identifies candidate molecules with potential as targets for innovative control strategies. Because controlling nymphs reduces subsequent adult populations, insights gained here may contribute to the development of sustainable approaches for managing spittlebug infestations in pasture systems.

## MATERIALS AND METHODS

### Insect material, RNA extraction and quality assessment

Fifth‐instar nymphs were identified based on body size and, primarily, on the advanced development of the wing pads, which extended beyond the second abdominal segment, according to established morphological criteria for Cercopidae (Marucci et al., 1999).

Nymphs of *Mahanarva spectabilis* were collected from naturally infested forage grass fields located at the experimental station of Embrapa Dairy Cattle (Coronel Pacheco, MG, Brazil). Nymphs were found at the basal portions of host plants (*Cenchrus purpureus* (syn. *Pennisetum purpureum*) cultivar BRS Capiaçu), enclosed within their characteristic foam masses and were feeding on xylem sap from forage grasses at the time of collection. After collection, insects were immediately processed or flash‐frozen in liquid nitrogen and stored at −80°C until RNA and protein extraction. No artificial feeding or laboratory rearing was performed prior to sample preparation.

For RNA extraction, 50 mg of insect tissue was homogenized in TRIzol (Invitrogen, Carlsbad, CA, USA), and RNA was isolated according to the manufacturer's instructions, including phase separation, RNA precipitation with isopropanol, washing with 75% ethanol and resuspension in nuclease‐free water. RNA concentration and purity were evaluated with a NanoDrop 2000 spectrophotometer (Thermo Scientific), and integrity was checked using an Agilent 2100 Bioanalyzer (Agilent Technologies, Santa Clara, CA, USA). Only high‐quality RNA with RNA integrity number (RIN) >7.0 and OD260/280 between 1.9 and 2.1 was used for library construction.

### Library construction and Illumina sequencing

A single RNA‐seq dataset was generated from a pooled sample comprising eight whole *M. spectabilis* nymphs to generate a comprehensive reference transcriptome.

Poly(A) + mRNA was enriched from total RNA using Oligo(dT)‐attached magnetic beads, fragmented into short fragments (200–300 bp) and used as templates for cDNA synthesis. First‐strand cDNA was synthesized using random hexamer primers, and RNase H was employed during second‐strand cDNA synthesis to remove RNA templates following first‐strand synthesis, in accordance with standard Illumina library preparation protocols. Second‐strand cDNA synthesis was completed using DNA polymerase I. After purification, cDNA fragments underwent end repair, A‐tailing and adapter ligation. Ligated products were size‐selected, PCR‐amplified and purified to generate cDNA libraries. Library quality and insert size distribution were verified on a Qsep400 (BiOptic Inc.), quantified by qPCR and sequenced on the Illumina NovaSeq 4000 platform (paired‐end 150 bp reads, PE150) (Illumina Inc., San Diego, CA, USA).

### Data processing and quality control

Raw reads were processed with Trimmomatic v0.39 (Bolger et al., 2014) to remove adapter sequences, low‐quality bases and reads containing more than 10% ambiguous nucleotides (N). Read quality before and after trimming was assessed using FastQC v0.11.9 (Andrews, 2010). Quality filtering was performed under the Phred33 encoding based on Q20, Q30 and GC content.

### De novo transcriptome assembly

High‐quality reads were assembled de novo using Trinity v2.8.5 (Grabherr et al., 2011), which applies a de Bruijn graph strategy. Briefly, Trinity consists of three sequential modules: (i) Inchworm, which assembles RNA‐seq data into the unique sequences of transcripts; (ii) Chrysalis, which clusters contigs and builds de Bruijn graphs for each cluster and (iii) Butterfly, which processes the graphs to generate full‐length transcripts and isoforms. The longest isoform per cluster was designated as a unigene. The quality control for assembly was assessed using metrics such as N50, mean transcript length and the total number of assembled transcripts.

The completeness of the *M. spectabilis* de novo transcriptome assembly was evaluated using BUSCO v6.0.0 (Benchmarking Universal Single‐Copy Orthologs) (Simão et al., 2015). The analysis was performed in transcriptome mode using the insecta_odb12 dataset, which comprises 3114 conserved orthologs representative of Insecta. BUSCO was run with default parameters and multithreading enabled to assess the proportion of complete (single‐copy and duplicated), fragmented and missing orthologs, providing an estimate of the overall quality and completeness of the assembled transcriptome.

### Expression quantification and analysis

Raw sequencing reads were first subjected to quality filtering to generate clean reads. This process included the removal of adapter‐contaminated reads, reads containing more than 10% ambiguous nucleotides (N), and reads in which more than 50% of bases had a Phred quality score ≤10. The resulting high‐quality clean reads were retained for downstream analyses.

Clean reads were aligned back to the assembled unigene set using Bowtie2 v2.3.4.1 (Langmead & Salzberg, 2012) with default parameters. Gene expression levels were subsequently estimated using RSEM v1.3.1 (Li & Dewey, 2011), which calculates transcript abundance based on the number of mapped read fragments. Expression values were normalized as FPKM (Fragments Per Kilobase of transcript per Million mapped reads), a metric that corrects for both transcript length and sequencing depth.

The distribution of unigene expression levels was assessed using density plots and boxplots to evaluate overall expression patterns and data consistency across the transcriptome.

### Signal peptide and secretion prediction

Predicted protein sequences used in this analysis were derived from open reading frames identified in the unigene set obtained from the transcriptome assembly. The selection of candidate sequences for secretome analysis was guided by proteomic evidence obtained from LC–MS/MS analyses of Batelli gland‐derived samples. Specifically, transcripts with corresponding protein identifications were prioritized, ensuring a higher confidence in their expression in the Batelli gland. These predicted proteins were generated by in silico translation of unigene‐derived coding sequences. The resulting protein sequences were used as input in FASTA format for signal peptide and secretion pathway prediction, as described below.

Predicted protein sequences from the Batelli gland dataset were used as input in FASTA format. Prior to analysis, sequences were quality‐checked by removing terminal stop characters (‘*’), converting non‐standard amino acids to canonical symbols when possible and excluding fragments <18 amino acids. When multiple genes/proteins shared the same functional annotation (new genome assembly), a single representative was retained by prioritizing (i) longest protein length and (ii) highest BLASTP bit score against UniProt to favour the most complete isoform.

Signal peptide (SP) presence and the putative Sec/SPI (Sec‐dependent signal peptide, Signal Peptidase I) cleavage site were predicted using SignalP 6.0 with the eukaryote model and default settings (Teufel et al., 2022).

In parallel, topology and additional N‐terminal signal sequences were inferred with Phobius v1.01 (long output), which jointly models signal peptides and transmembrane helices. For each protein, the SignalP output (‘SP’ vs. ‘OTHER’) and the most probable cleavage position were recorded, together with Phobius calls for SP (Y/N) and the number of predicted transmembrane (TM) helices.

We then applied a simple, transparent consensus to classify export likelihood. Proteins were labelled *high* confidence if they were SignalP‐positive (Sec/SPI) and either (a) Phobius also predicted an SP or (b) Phobius predicted ≤1 TM helix overall (allowing for a single TM attributable to the signal peptide). Proteins were labelled *medium* confidence if at least one tool (SignalP or Phobius) indicated an SP but the *high* criteria were not met. All remaining proteins were labelled *low*. Predicted cleavage sites reported by SignalP were kept as the operative signal peptide cuts for subsequent analyses.

To build the working ‘secretome’ set, we retained all proteins classified as SignalP‐positive and/or consensus *medium*/*high* and removed clear multi‐pass membrane proteins (≥2 TM helices beyond the signal peptide region). The resulting non‐redundant set was ranked by (1) consensus tier (*high* > *medium*), (2) presence of a well‐defined cleavage site, (3) absence of additional TM segments, (4) protein length and (5) BLASTP bit score.

### Alternative splicing analysis

To assess transcriptome complexity arising from alternative splicing (AS), we leveraged the isoform structure generated by the de novo transcriptome assembly performed with Trinity v2.15.1 (Grabherr et al., 2011). In the Trinity nomenclature, transcripts sharing the same ‘gene’ identifier (e.g., TRINITY_DNxxxxx_cY_gZ) but distinct isoform suffixes (i1, i2, etc.) are considered isoforms of a single unigene cluster. The transcript FASTA file (Demo.Transcripts.fa) was parsed using custom Python 3.10 scripts to count isoforms per Trinity gene. For each gene, isoform counts were computed, frequency distributions generated and descriptive statistics (total number of genes, maximum and average isoforms per gene and proportion of multi‐isoform loci) calculated. Genes with ≥2 isoforms were defined as multi‐isoform loci and considered potential AS candidates.

To integrate AS information with functional annotation, the isoform dataset was merged with Pfam, KEGG and expression results. Pfam domains were identified using HMMER v3.3.2 against the Pfam‐A database (Mistry et al., 2021). KEGG orthologs and pathways were assigned via KAAS (Moriya et al., 2007), while unigene expression values were estimated as FPKM from RNA‐seq read alignments using Bowtie2 v2.4.5 (Langmead & Salzberg, 2012) and RSEM v1.3.3 (Li & Dewey, 2011). Genes were classified into functional categories relevant to host–environment interactions (Detoxification, Chemosensory, Osmoregulation/Transport, Cuticle/Barrier, Stress response, Immune defence and Plant digestion/interaction) by applying curated regular expression filters to Pfam and KEGG annotation fields. For each category, the number and percentage of genes with AS was calculated relative to the total annotated set.

### Proteomic analysis of Batelli gland by LC/MS

Proteomic analysis was specifically performed on the Batelli gland, a specialized secretory organ that plays a central role in foam production and is essential for the protection and survival of *M. spectabilis* nymphs.

#### Protein extraction and in‐solution digestion

Batelli glands were carefully dissected from *M. spectabilis* nymphs. Briefly, the thorax was separated from the abdomen, and the abdominal cuticle was longitudinally opened using a sterile scalpel under a stereomicroscope (Olympus HZS10) with a 0.75× objective. The Batelli glands were then manually removed from the abdominal cavity using fine forceps and immediately transferred to acidified water (pH 3.0) to preserve glandular integrity and minimize protein degradation. Approximately 30 glandular segments obtained from ten fifth‐instar nymphs were pooled and subjected to cell lysis by sonication in SP3 lysis buffer (100 mM triethylammonium bicarbonate [TEAB], 1% sodium dodecyl sulfate [SDS], 10 mM tris(2‐carboxyethyl)phosphine [TCEP] and 40 mM chloroacetamide [CAA]), following the Single‐Pot Solid‐Phase‐enhanced Sample Preparation (SP3) protocol (Hughes et al., 2018). Following lysis, insoluble debris was removed by centrifugation, and the clarified supernatant was collected for protein quantification using the bicinchoninic acid (BCA) assay. Proteins were subsequently digested with trypsin using magnetic beads, according to the SP3 workflow, to generate peptides for downstream mass spectrometric analysis.

#### Protein identification by LC–MS/MS

The peptides were dissolved in 50 μL of 0.1% formic acid and analysed using a nano‐UHPLC system (UltiMate R 3000, Dionex, San Jose, USA) equipped with a Acclaim PepMap100 C18 Nano‐Trap column (100 μm i.d. × 20 mm, 5 μm, 100 Å; Thermo Scientific, Waltham, MA, USA) and an analytical column using un Acclaim PepMap100 C18 RSLC column (75 μm i.d. × 150 mm, 2 μm, 100 Å; Thermo Scientific, Waltham, MA, USA), in tandem with the trap column operating at a constant flow rate of 0.3 μL min^−1^. The solvent used was: (A) 0.1% formic acid and (B) 80% acetonitrile, 0.1% formic acid. The multistep gradient was applied as follows: a conditioning step with 3.8% of B during 3 min, followed by a ramp from 3.8 to 30% B over 120 min, and subsequently, 30%–55% B to 150 min. Then a final ramp with 99% B to 162 min, followed by a reconditioning step with 3.8% B until 180 min.

The spectral data was acquired using a Q‐Exactive mass spectrometer (Thermo Scientific, Bremen, Germany) operating at full‐scan/MS2 mode. The nanospray flex ion source (Thermo Scientific) was set at 3.8 kV on positive mode, with a capillary temperature of 250°C and S‐lens levels set to 55. The Data Dependent Acquisition method (DDA) was performed in the top 12 ions with charge states between +2 and + 4 from a 1.2 m/z window. Survey scans were tuned for a resolution of 70,000 with a mass range between 300 and 2000 m/z. For MS/MS scans, selected ions were fragmented by Higher Energy Collision Dissociation (HCD) with a stepped Normalized Collisional Energy (NCE) of 28–30. After the dissociation, the fragmentation spectra were acquired using a resolution of 17,500 performing a maximum injection time of 60 ms with a dynamic exclusion time of 40 s and a target value of 5e^5^ (minimum AGC target 6.25e^3^).

The raw data were processed using PEAKS Studio v12.5 (Bioinformatics Solutions Inc., Waterloo, ON, Canada), which performed protein identification through a combined approach of de novo sequencing and database searching. Database searches were performed against the unigene database obtained from transcriptome analysis of *M. spectabilis* nymphs (Sections 2.4 and 2.5).

The parameters used in the program were: unigenes from *M. spectabilis* as the reference database; methionine oxidation as a variable modification; and cysteine carbamidomethylation as a fixed modification; one missed cleavage, charge states of 2+, 3+ and 4+; trypsin‐*like* cleavage enzyme, and a mass error of 20 ppm. For confident identification, the false discovery rate (FDR) was considered to be <0.5% for peptides and proteins identified using an FDR < 1% and showing at least 2 unique peptides.

## RESULTS

### Sequencing quality and assembly statistics of RNA‐seq data

Illumina sequencing of the *M. spectabilis* nymph transcriptome generated a total of 63,010,306 raw reads with high base quality (Q30 = 94.09%), corresponding to 18.86 Gb of high‐quality bases. Raw reads were processed to remove adaptors and low‐quality sequences, generating clean FASTQ files, deposited in the NCBI Sequence Read Archive (SRA) under BioProject PRJNA1404840. The overall GC content was 39.06%, and 94.09% of the bases reached Q30 or higher, indicating that the dataset was of excellent quality with very low base‐calling error rates. Indeed, base quality assessment showed that Q20, Q30 and Q40 corresponded to accuracies of 99%, 99.9% and 99.99%, respectively, confirming the reliability of the sequencing run.

The average error rate along reads was extremely low (<0.0003), with a slight increase toward the end of both Read1 and Read2, which is consistent with typical Illumina sequencing profiles (Figure S1). Importantly, no abrupt quality deterioration was observed, indicating reliable base calling across the full read length. Base composition analysis showed a nearly uniform distribution of nucleotides after the initial cycles, where minor fluctuations are expected due to random priming effects (Figure S2). After position ~20, the proportions stabilized, with adenine (A) and thymine (T) comprising ~30% each, and guanine (G) and cytosine (C) representing ~20% each. This balanced representation reflects the absence of systematic nucleotide bias and suggests that the sequencing library was of high complexity. The proportion of undetermined bases (N) was negligible throughout the reads. These metrics indicate that the sequencing of the *M. spectabilis* nymph sample produced high‐quality reads suitable for downstream transcriptome assembly and gene expression analyses.

To evaluate the quality and representativeness of the sequencing library, several diagnostic analyses were performed (Figure S4). Randomness assessment of read distribution across transcripts demonstrated uniform coverage from the 5′ to the 3′ ends of genes, with only a slight enrichment in the central regions (Figure S4A). This pattern indicates that the library construction was unbiased and that reads were evenly distributed along transcripts. Insert size distribution analysis revealed that the majority of paired‐end fragments ranged between 300 and 500 bp, with a clear peak at approximately 400 bp (Figure S4B). This insert size is consistent with the expected library preparation.

Gene saturation analysis, using fragments per kilobase of transcript per million mapped reads (FPKM ≥0.1), showed that the number of detected genes plateaued after ~10 million reads, reaching nearly 100,000 transcripts (Figure S4C). This result indicates that the sequencing depth was sufficient to capture the majority of expressed genes in the nymph stage of *M. spectabilis*.

### De novo assembly and mapping statistics of *Mahanarva spectabilis* nymph transcriptome

After quality filtering, 63.5% of reads (40,015,826) were successfully mapped to the assembled unigenes, of which approximately half (49.1%) mapped uniquely, while the remaining 50.9% were multi‐mapped.

The de novo transcriptome assembly produced a comprehensive unigene dataset with transcripts ranging from short fragments to full‐length sequences. In total, 100,666 unigenes were assembled. The length distribution showed that the majority of unigenes fell within the 300–1000 nt range (64.4%), while 13.9% were between 1000 and 2000 nt, and a significant fraction (8.6%) exceeded 2000 nt (Figure S3). These longer unigenes likely represent full‐length or nearly complete transcripts, which are particularly valuable for functional annotation and downstream analyses. A substantial fraction of longer unigenes (>3000 nucleotides) was also recovered, reflecting the presence of full‐length or nearly full‐length transcripts.

The de novo assembly resulted in 99,669 unigenes, with a unigene N50 of 1259 bp (transcript N50 = 1598 bp) and a mean unigene length of 878 bp, indicating good assembly continuity and representation.

The completeness of the *M. spectabilis* transcriptome assembly was assessed using BUSCO with the insecta_odb12 lineage dataset. The analysis recovered 89.2% complete BUSCOs, of which 88.4% were single‐copy and 0.8% were duplicated. In addition, 2.9% of BUSCOs were classified as fragmented, while 7.9% were missing. These results indicate a high level of completeness and low redundancy, supporting the overall quality and completeness of the de novo assembly.

### Functional annotation of unigenes

Functional annotation of unigenes was performed based on sequence homology and protein domain identification. Unigene sequences were searched against the NR, SwissProt, COG, KOG, eggNOG (v4.5) and KEGG databases using DIAMOND. KEGG Orthology assignments were obtained using KOBAS. Gene Ontology (GO) terms were assigned using InterProScan based on conserved protein domains and integrated functional signatures. No Gene Ontology enrichment analysis was performed in this study; GO terms were used solely for functional classification and descriptive analyses of the annotated transcriptome.

To gain insights into the biological functions of the assembled unigenes, multiple functional databases were queried, including COG, GO, KEGG, KOG, Pfam, eggNOG, NR and SwissProt. In total, a substantial fraction of unigenes could be successfully annotated, highlighting the robustness of the dataset for downstream analyses. Among the annotation resources, GO (Gene Ontology) provided functional information for 13,282 unigenes, while KEGG (Kyoto Encyclopedia of Genes and Genomes) assigned 10,768 unigenes to metabolic and signalling pathways. Pfam domain searches resulted in 12,945 matches, and KOG (Eukaryotic Orthologous Groups) classification identified 8404 unigenes. Additionally, COG (Clusters of Orthologous Groups) analysis annotated 4958 unigenes, reflecting conserved orthologous functions across species.

**FIGURE 1:**
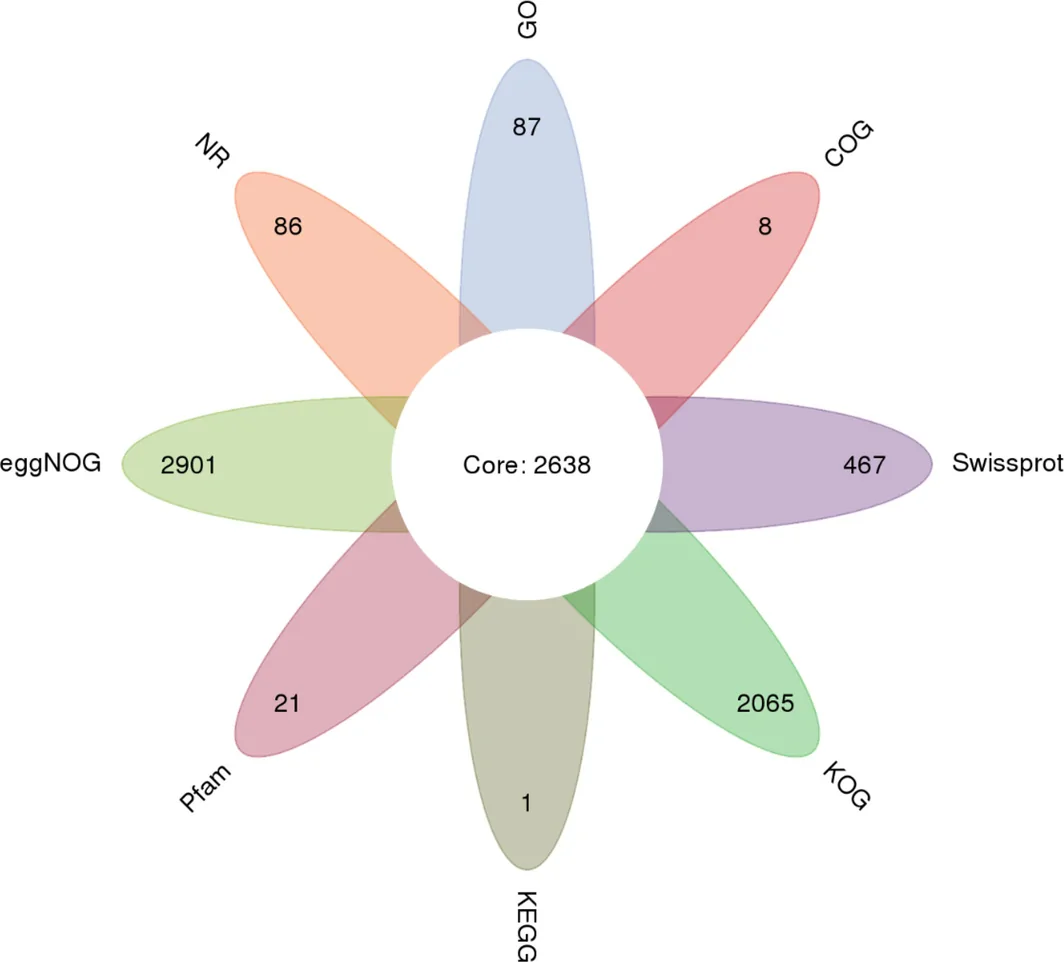
Functional annotation of *Mahanarva spectabilis* nymph unigenes across multiple databases. The flower plot illustrates shared among all and unique annotations, with a core set of 2638 unigenes functionally characterized across multiple databases.

The integrative ‘flower plot’ representation (Figure 1) illustrates the overlap among annotation databases. A core set of 2638 unigenes was consistently annotated across multiple databases, underscoring their strong functional conservation. Beyond the core, additional unique annotations were obtained from individual resources, such as 2901 unigenes exclusively annotated in eggNOG, 2065 in NR, and 467 in SwissProt, among others. These results indicate that a large proportion of the *M. spectabilis* nymph transcriptome could be functionally characterized, providing a valuable foundation for gene function exploration and pathway analysis in this developmental stage. Given the absence of a reference genome for *M. spectabilis*, the identification of 2638 unigenes consistently annotated across multiple databases represents a substantial and biologically meaningful fraction of the transcriptome, reflecting a robust core of conserved and functionally supported genes.

### Overview of general biological functions in the *M. spectabilis* nymph transcriptome

**FIGURE 2:**
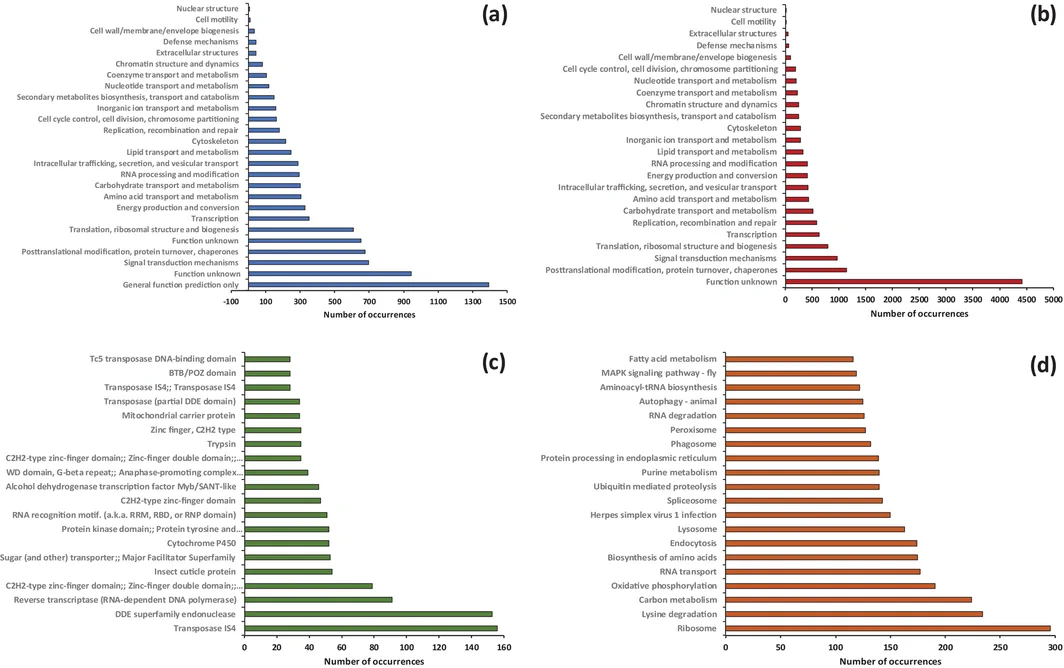
Functional annotation of unigenes from the *Mahanarva spectabilis* nymph transcriptome. (a) KOG functional classification of unigenes. The largest categories were general function prediction only, followed by signal transduction mechanisms and posttranslational modification, protein turnover and chaperones. (b) eggNOG functional classification of unigenes. The predominant category was function unknown, while a substantial number of transcripts were involved in posttranslational modification, signal transduction, translation and ribosomal structure, and transcription. (c) Top 20 Pfam domains identified across the assembled unigenes. The most frequent protein domains included transposase IS4, DDE superfamily endonuclease, reverse transcriptase (RNA‐dependent DNA polymerase), C2H2‐type zinc‐finger domains and insect cuticle proteins, reflecting the diversity of structural and enzymatic motifs in the transcriptome. (d) Top 20 KEGG pathways represented in the *M. spectabilis* nymph transcriptome. The Table S2 lists the most enriched pathways, including the KEGG identifier and the number of unigenes assigned to each pathway. Highly represented categories included ribosome biogenesis, amino acid metabolism, oxidative phosphorylation, RNA transport and central carbon metabolism, highlighting the strong representation of fundamental cellular and metabolic processes in the nymph stage.

To obtain a comprehensive functional overview of the *M. spectabilis* nymph transcriptome, assembled unigenes were annotated against multiple reference databases, including eggNOG/COG/KOG, Pfam, KEGG, GO, SwissProt and NR (Figure 2 and Table S1). Each database provided complementary layers of information, allowing both broad and fine‐grained functional characterization.

For instance, Pfam captured domain‐level signatures not detected in KOG/eggNOG, while eggNOG/KOG provided phylogenetically conserved functional predictions that contextualize insect‐specific adaptations. These annotations confirm that the nymph transcriptome of *M. spectabilis* encodes a broad repertoire of genes involved in essential biological processes, signalling pathways and metabolic functions while also uncovering a large set of transcripts with unknown functions that may represent novel genes or lineage‐specific innovations.

The Pfam database identified thousands of conserved protein domains, including peptidase families, transporters, transcription factors and RNA‐binding proteins, highlighting the diversity of structural and catalytic motifs encoded in the transcriptome. The most abundant domains included transposase IS4, DDE superfamily endonuclease, reverse transcriptase (RNA‐dependent DNA polymerase), C2H2‐type zinc‐finger domains and insect cuticle proteins, reflecting both housekeeping and insect‐specific gene repertoires (Figure 2c).

Classification through eggNOG and KOG assigned unigenes into major functional categories, including metabolism, cellular processes, information storage and a ‘general function prediction’ class, which comprises genes with conserved features but without a clearly defined specific function. In KOG, the most abundant groups corresponded to ‘general function prediction only’, signal transduction mechanisms and posttranslational modification, protein turnover and chaperones (Figure 2a,b). In eggNOG, the predominant category was ‘function unknown’, followed by transcripts involved in posttranslational modification, signal transduction, translation and ribosomal structure and transcription (Figure 2c). The high proportion of unigenes classified as having unknown or general functions is a common feature of transcriptomes from non‐model insects and reflects the presence of conserved genes whose specific biological roles remain poorly characterized, alongside a broad representation of essential cellular and regulatory processes.

Integration across annotation resources revealed a core set of transcripts annotated by multiple databases, strengthening the confidence in functional assignment. At the same time, each individual resource contributed unique information. For example, Pfam captured domain‐level signatures not detected in KOG/eggNOG, while eggNOG/KOG provided phylogenetically conserved functional predictions that contextualize insect‐specific adaptations.

KEGG annotation revealed that 8836 unigenes were mapped to metabolic and signalling pathways, providing a broad overview of the functional landscape of the *M. spectabilis* nymph transcriptome (Figure 2d). The most represented categories were associated with primary metabolism, particularly energy and carbohydrate metabolism. Among these, glycolysis/gluconeogenesis (103 unigenes), the citrate cycle (TCA cycle; 77 unigenes), and the pentose phosphate pathway (58 unigenes) were the most enriched, underscoring the strong representation of central carbon metabolism.

In addition to carbohydrate metabolism, several pathways related to amino acid biosynthesis and degradation, lipid metabolism and cofactor/vitamin biosynthesis were also highly represented. Notably, unigenes were also assigned to pathways involved in signal transduction and environmental information processing, including genes related to MAPK and PI3K‐Akt signalling cascades, which are commonly associated with cellular stress responses and insect adaptation to host plants.

### Transcriptomic signatures underlying plant interaction and environmental adaptation in *M. spectabilis* nymphs

Our transcriptome survey revealed a broad repertoire of genes classified by functional annotation as being potentially involved in plant–insect and environment–nymph interactions (Tables 1, S2 and S3). These inferences are based on conserved Gene Ontology terms, Pfam domains and KEGG pathways, and do not imply experimental validation of their roles in *M. spectabilis*. Collectively, these categories comprise approximately 6%–7% of the total unigene dataset and represent a biologically targeted subset selected for their relevance to nymph survival, foam‐mediated protection and plant‐environment interactions.

**TABLE 1:** Summary of transcript categories identified in the *Mahanarva spectabilis* nymphal transcriptome related to host–plant interaction and environmental adaptation.

Category	PFAM hits	KEGG hits	Overlap
Detoxification	317	217	96
Chemosensory	11	18	0
Plant interaction digestion	65	0	0
Stress response	218	0	0
Osmoregulation transport	42	100	20
Cuticle barrier	65	0	0
Immune defence	43	33	4

Detailed gene lists are provided in Supplementary Tables S2 and S3.

Genes involved in the detoxification of plant secondary metabolites and xenobiotics were strongly represented. A total of 317 transcripts carried typical Pfam domains, including cytochrome P450 monooxygenases (CYP450s), glutathione S‐transferases (GSTs), UDP‐glycosyltransferases (UGTs), carboxylesterases and short‐chain/aldo‐keto reductases (SDR/AKR). In parallel, 217 KEGG annotations were associated with detoxification‐related functions, with 96 transcripts overlapping both Pfam and KEGG, indicating robust support for detoxification capacity. Enriched KEGG pathways included Glutathione metabolism (ko00480; 80 genes), Metabolism of xenobiotics by cytochrome P450 (ko00980; 39 genes), Drug metabolism via P450 (ko00982; 36 genes) and Drug metabolism by other enzymes (ko00983; 100 genes). Among the most highly expressed candidates were ABC transporters, GSTs, carboxylesterases and CYP450 monooxygenases.

Transcriptomic signatures related to chemosensation, essential for host–plant recognition and habitat selection, were also detected. Pfam and KEGG analyses recovered 11 and 18 chemosensory‐related transcripts, respectively, including odorant‐binding proteins (OBPs), pheromone‐binding proteins (PBPs), chemosensory proteins (CSPs) and receptors from the OR/GR/IR families. KEGG additionally identified two genes associated with the Olfactory transduction pathway (ko04740). Notably, the most highly expressed chemosensory candidates belonged to the PBP/GOBP family.

Genes associated with osmoregulation and ion transport, which are critical for xylem‐feeding insects, were highly abundant. We identified 42 Pfam and 100 KEGG hits corresponding to V‐ATPases, Na^+^/K^+^‐ATPases, solute carrier (SLC) transporters and carbonic anhydrases, with 20 transcripts supported by both annotation systems. Multiple V‐ATPase subunits, E1–E2 ATPases and carbonic anhydrases ranked among the most highly expressed transcripts, reflecting the physiological demands imposed by a dilute, water‐rich diet.

Environmental adaptation was further supported by transcripts involved in cuticle formation and remodelling. A total of 158 transcripts encoded cuticular proteins (CPR families RR‐1, RR‐2, CPF, CPLCG) and chitin metabolic enzymes, including chitinases, chitin deacetylases and laccases (Tables 1, S2 and S3). These gene families are essential for the formation of protective barriers and likely contribute to resistance against desiccation and microbial invasion within the foam environment.

Finally, transcripts related to stress responses and innate immunity were abundant. Pfam annotation identified 218 transcripts encoding heat shock proteins (HSPs) and antioxidant enzymes such as superoxide dismutases, catalases and peroxiredoxins. Components of innate immune pathways were also represented, with 43 Pfam and 33 KEGG hits encompassing serine proteases/serpins, lysozymes and pathogen recognition receptors, including PGRP and Toll pathway elements. Together, these gene sets highlight molecular mechanisms that support survival, stress tolerance and defence against plant‐associated microorganisms.

### Alternative splicing predictions in the nymphal transcriptome

Alternative splicing patterns reported here are based on transcriptome‐wide computational predictions derived from de novo assembly and represent estimates of isoform diversity rather than experimentally validated splicing events.

The de novo transcriptome assembly of *M. spectabilis* nymphs revealed a substantial degree of isoform diversity. Based on Trinity clustering, a total of 99,669 unigene clusters were identified, of which 16,374 (16.43%) produced at least two isoforms, indicative of alternative splicing (AS). The average number of isoforms per unigene was 1.40, with a maximum of 75 isoforms observed in a single locus. The distribution of isoform counts was strongly right‐skewed (Tables [Link], [Link]), with the majority of genes presenting a single isoform and progressively fewer loci carrying multiple isoforms. Isoform status indicated that ~84% of unigene clusters were single‐isoform, whereas ~16% were multi‐isoform.

**FIGURE 3:**
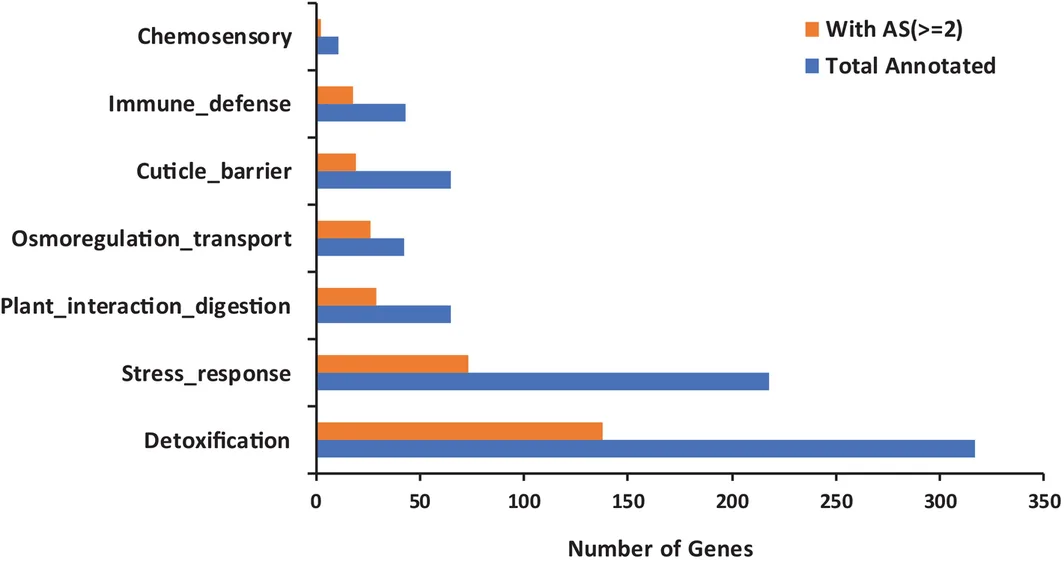
Genes with and without alternative splicing across functional categories in the *Mahanarva spectabilis* nymph transcriptome. Barplot showing the number of annotated genes in each functional category classified as single‐isoform (blue, ‘Without AS’) or multi‐isoform (orange, ‘With AS ≥2’). Categories include detoxification, chemosensory, osmoregulation/transport, cuticle/barrier, stress response, immune defence and plant interaction/digestion. A substantial proportion of detoxification and stress‐related genes exhibit multiple isoforms, highlighting the contribution of alternative splicing to functional diversification in nymphal stages.

To evaluate whether AS preferentially affected specific functional gene families, multi‐isoform loci were cross‐referenced with Pfam and KEGG annotations and grouped into categories relevant to host–plant and host–environment interactions (Figure 3, Table S6). The analysis revealed that several functionally important categories harboured a notable proportion of alternative splicing genes. Detoxification‐related transcripts (cytochrome P450s, GSTs, UGTs, carboxylesterases and ABC transporters) exhibited extensive isoform diversity, consistent with their role in metabolic plasticity and xenobiotic processing. Similarly, chemosensory genes (odorant/gustatory/ionotropic receptors, OBPs and CSPs) and osmoregulatory transporters (V‐ATPases, aquaporins and ion exchangers) also showed multiple isoforms, suggesting isoform‐level modulation of host detection, water balance and homeostasis. Multi‐isoform cuticle proteins, stress‐responsive factors (heat‐shock proteins, antioxidants) and immune‐related genes (lysozymes, PGRPs, serine proteases, defensins) were also abundant, reflecting the contribution of alternative splicing to physiological adaptation and defence mechanisms. Finally, plant–interaction enzymes (cellulases, pectinases, glycosidases, amylases) also included several multi‐isoform loci, pointing to potential isoform‐level specialization in plant cell wall digestion.

### Secretome prediction (SignalP and Phobius) from RNA‐seq analysis

Signal peptide prediction was performed on the full set of proteins predicted from the transcriptome assembly (Table S7). SignalP identified 168 proteins as candidates for secretion, and Phobius independently confirmed secretion signals in 147 of these candidates. The strong concordance between predictors supports their classification as putative secreted proteins.

This set of secreted proteins includes enzymes (e.g., oxidoreductases, protein disulfide isomerases), chaperones (e.g., endoplasmic reticulum BiP), structural glycoproteins (e.g., cuticle‐related proteins) and storage proteins (e.g., hexamerins), all of which may contribute to the biochemical composition of the protective foam secreted by the gland.

### Functional annotation of the predicted secretome from RNA‐seq analysis

**FIGURE 4:**
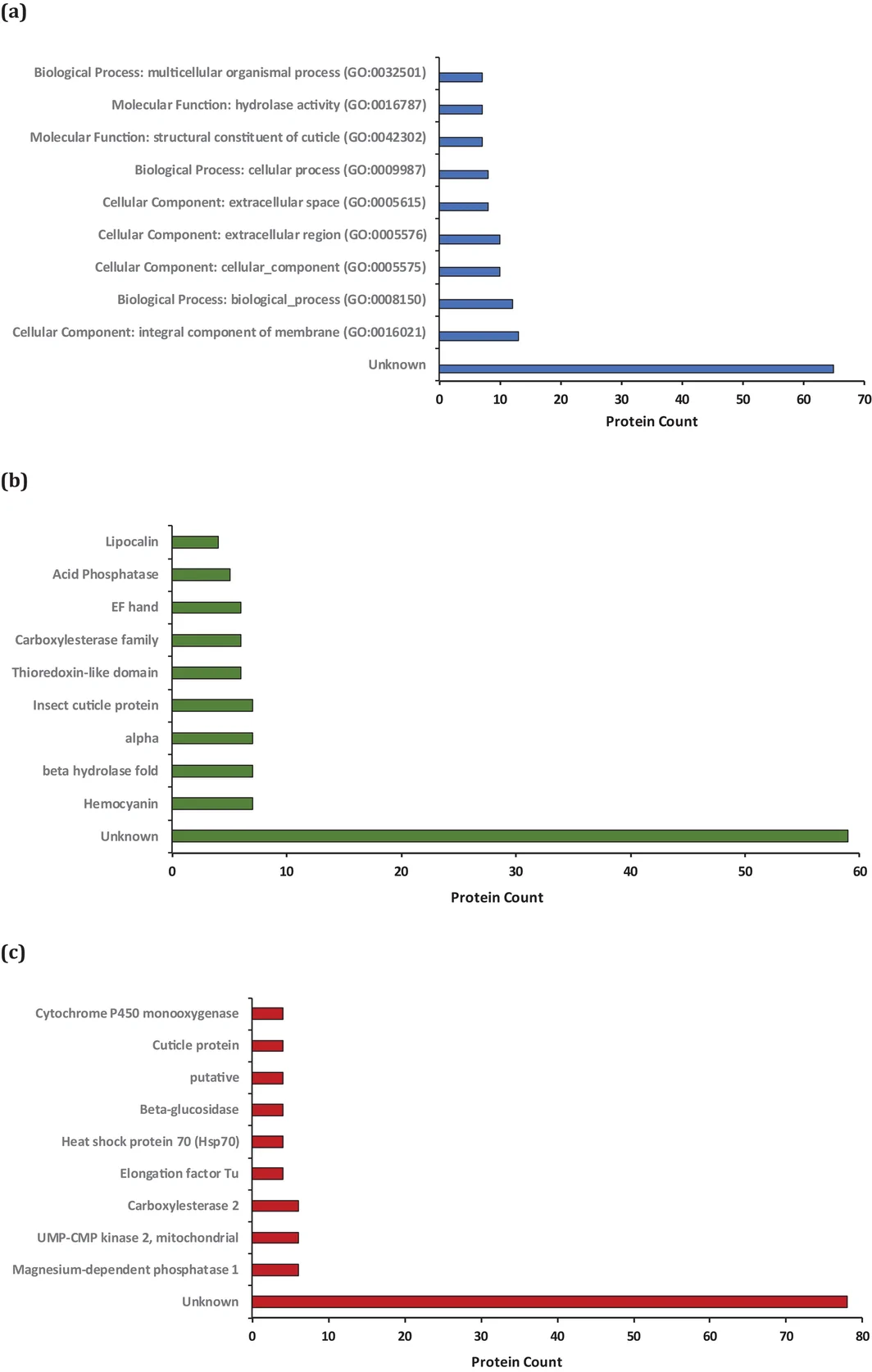
Top functional annotations of the secretome based on Pfam (b), KEGG (a) and Gene Ontology (c). The bar chart displays the ten most represented categories retrieved from each database, with KEGG identifiers replaced by descriptive functional names. Pfam highlights included hemocyanin‐like domains, beta‐hydrolase folds and insect cuticle protein motifs. KEGG annotations recovered enzymes such as cytochrome P450 monooxygenases, beta‐glucosidases and carboxylesterases, as well as structural and transport proteins. GO terms were enriched for integral membrane components, extracellular region and catalytic activities. Despite the broad functional coverage provided by Pfam, KEGG and GO, a substantial subset of proteins remained without functional annotation (Table S8). Specifically, 59 proteins (Pfam), 78 proteins (KEGG) and 65 proteins (GO) lacked any assigned domain, pathway or ontology term, being classified as ‘unannotated.’ These results indicate that approximately one third of the predicted secretome corresponds to proteins with unknown or poorly characterized functions.

Functional classification of the 168 secreted proteins based on GO, KEGG and PFAM revealed enrichment in categories related to extracellular localization, structural functions and enzymatic activity (Figure 4 and Table S8). The GO annotation highlighted terms related to membrane and extracellular localization, with ‘integral component of membrane’ (13 proteins) and ‘extracellular region’ (10 proteins) among the most represented categories (Table 2). Functional assignments also included proteins associated with metabolic processes, ATP binding and general catalytic activities, supporting a role of the secretome in extracellular enzymatic activity and molecular interactions. The KEGG analysis retrieved multiple enzyme families, including K17619 (6 proteins), K13809 (6 proteins) and K03927 (6 proteins), which are linked to redox metabolism and protein processing pathways (Table 2). Additional annotations were assigned to enzymes involved in detoxification and transport, consistent with the requirement of the gland to manage plant‐derived compounds and contribute to foam matrix composition.

**TABLE 2:** Top functional categories of the predicted secretome from RNA‐seq analysis of *Mahanarva spectabilis*.

Term	Count	Source
Unknown	65	GO
Cellular Component: integral component of membrane (GO:0016021)	13	GO
Biological Process: biological process (GO:0008150)	12	GO
Cellular Component: cellular component (GO:0005575)	10	GO
Cellular Component: extracellular region (GO:0005576)	10	GO
Cellular Component: extracellular space (GO:0005615)	8	GO
Biological Process: cellular process (GO:0009987)	8	GO
Molecular Function: structural constituent of cuticle (GO:0042302)	7	GO
Molecular Function: hydrolase activity (GO:0016787)	7	GO
Biological Process: multicellular organismal process (GO:0032501)	7	GO
Unknown	78	KEGG
Magnesium‐dependent phosphatase 1	6	KEGG
UMP‐CMP kinase 2, mitochondrial	6	KEGG
Carboxylesterase 2	6	KEGG
Elongation factor Tu	4	KEGG
Heat shock protein 70 (Hsp70)	4	KEGG
Beta‐glucosidase	4	KEGG
putative	4	KEGG
Cuticle protein	4	KEGG
Cytochrome P450 monooxygenase	4	KEGG
Unknown	59	PFAM
Hemocyanin	7	PFAM
Beta‐hydrolase fold	7	PFAM
alpha	7	PFAM
Insect cuticle protein	7	PFAM
Thioredoxin‐like domain	6	PFAM
Carboxylesterase family	6	PFAM
EF hand	6	PFAM
Acid Phosphatase	5	PFAM
Lipocalin	4	PFAM

The table summarizes the most represented Gene Ontology (GO), KEGG and Pfam terms. The number of proteins associated with each annotation term is shown for comparison across databases.

The PFAM domain analysis revealed the predominance of structural and enzymatic motifs (Tables 2 and S8). Among them, hemocyanin‐like domains (7 proteins), beta‐hydrolase folds (7 proteins) and insect cuticle protein domains (7 proteins) were especially abundant, reinforcing the roles of oxygen‐binding glycoproteins, hydrolytic enzymes and structural proteins in the composition of the secretome.

The KEGG analysis (Tables 2 and S8) retrieved multiple enzyme families, including *magnesium‐dependent phosphatase 1* (K17619), *UMP‐CMP kinase 2, mitochondrial* (K13809) and *carboxylesterase 2* (K03927), each represented by several proteins. These enzymes are linked to redox metabolism, nucleotide metabolism, detoxification and protein processing pathways. Additional KEGG terms mapped include others involved in transport and extracellular secretion.

### Secreted proteins linked to host–plant interaction and environmental survival

The set of 168 high‐confidence secreted proteins included multiple functional classes potentially associated with plant–insect interaction and environmental survival (Table 3). Among these, detoxification‐ and redox‐related enzymes were represented by nine candidates, including carboxylesterases, glutathione‐related oxidoreductases and P450‐associated proteins, suggesting a role in the extracellular processing of plant‐derived compounds and xenobiotics.

**TABLE 3:** Predicted secreted proteins from the RNA‐Seq dataset of *Mahanarva spectabilis* nymphs were identified based on the presence of N‐terminal signal peptides.

Functional group	Secreted proteins	ProteinID
Chaperones / Protein folding	4	Gene_25378, Gene_35197, Gene_50668, Gene_99264
Chemosensory	1	Gene_126242
Cuticle / Structural	10	Gene_41268, Gene_38,689, Gene_53331, Gene_29966, Gene_31904, Gene_24102, Gene_34920, Gene_126386, Gene_56704, Gene_81905
Detoxification / Redox	9	Gene_47720, Gene_114391, Gene_79666, Gene_24447, Gene_23337, Gene_125559, Gene_100906, Gene_123132, Gene_78940
Immune / Antimicrobial	3	Gene_32042, Gene_89309, Gene_28231
Osmoregulation / Enzymes	8	Gene_28,076, Gene_18758, Gene_117898, Gene_47660, Gene_60582, Gene_64326, Gene_33482, Gene_6183
Proteases / Protease inhibitors	11	Gene_52229, Gene_17926, Gene_23192, Gene_177, Gene_125745, Gene_60582, Gene_42899, Gene_52512, Gene_25589, Gene_95455, Gene_46772
Storage / Lipocalins	5	Gene_32042, Gene_89309, Gene_27730, Gene_99181, Gene_76286

Proteins were grouped into functional categories relevant to host–plant interaction and environmental survival. The table lists the number of predicted secreted proteins in each category and the corresponding ProteinIDs.

Proteases and protease inhibitors were also prominent, with 11 signal peptide–positive proteins, including serine proteases and serpins. These proteins may contribute to extracellular matrix remodelling within the foam and to defence‐related processes. In addition, 10 secreted proteins were classified as cuticle or structural components, indicating a potential contribution to barrier formation or reinforcement at the foam‐insect interface.

Proteins associated with immune and antimicrobial functions were represented by three candidates with lysozyme‐ or phenoloxidase‐like signatures, consistent with protective roles in the foam microenvironment. Furthermore, eight secreted proteins were linked to osmoregulation and solute transport, including carbonic anhydrases and membrane‐associated transporters, reflecting physiological adaptations to the aqueous and ionically dilute environment characteristic of sap‐feeding insects.

The secretome also included four secretory‐pathway chaperones, such as protein disulfide isomerases and heat shock proteins, which may support folding and stability of exported proteins in the extracellular milieu. Finally, five secreted candidates were annotated as carrier or storage‐related proteins, potentially involved in buffering the chemical composition of the foam or interacting with plant‐derived molecules. A single signal peptide–positive odorant‐binding/chemosensory protein was detected, suggesting a possible role in binding volatile or semiochemical compounds at the plant–foam interface.

### LC–MS/MS identification and quantitative profiling of the Batelli gland proteome

Shotgun LC–MS/MS of the Batelli gland from nymphs of *M. spectabilis* yielded 30,270 MS and 14,444 MS/MS scans (Orbitrap MS with LIT MS/MS; nano‐ESI, high‐energy CID). Database searching in PEAKS (homology mode) and filtering (peptide −10lgP ≥ 17; protein −10lgP ≥ 20; ≥2 unique peptides; de novo ALC ≥50%) identified 8094 PSMs, 3806 distinct peptides and 500 proteins grouped into 493 protein groups, with FDRs of 0.5% (PSM), 0.8% (peptide) and 0.0% (protein). Most proteins (371) were supported by >2 unique peptides (129 with exactly 2). Quality controls showed predominantly fully tryptic peptides (3681 with zero missed cleavages; 125 with one), and 3650 spectra were classified as ‘de novo only’; precursor‐mass‐error and score distributions are reported in the quality control (QC) output.

### Functional annotation and biological interpretation of the Batelli gland proteome

Based on the proteins experimentally identified by LC–MS/MS in Section 3.10, we next examined their functional annotation and biological significance using PFAM, KEGG and Gene Ontology analyses.

**FIGURE 5:**
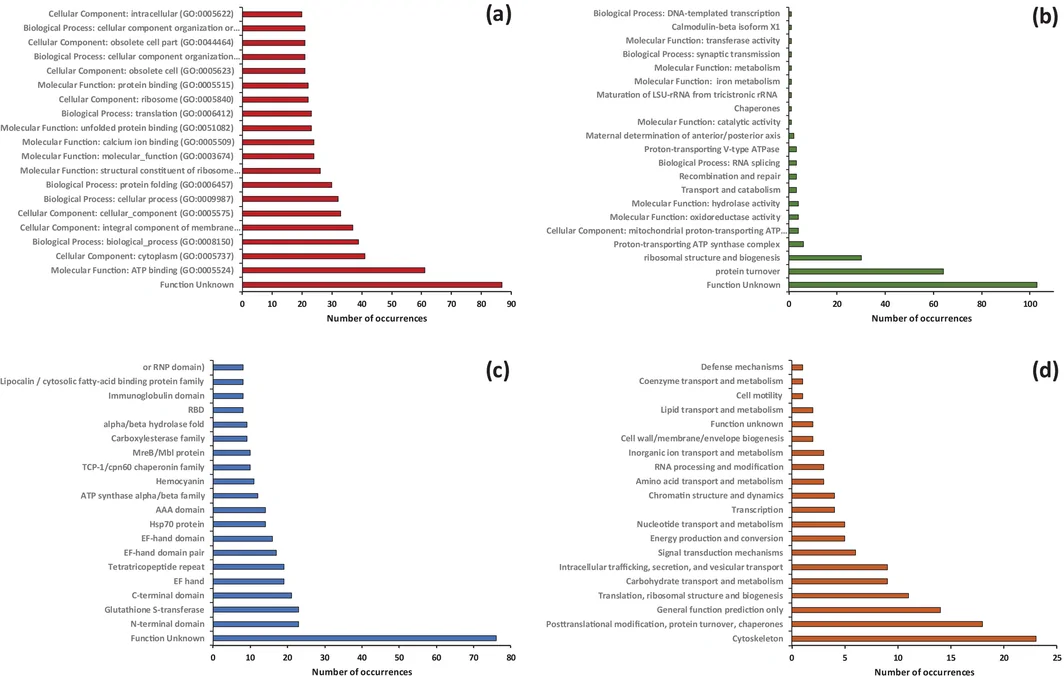
Functional classification of proteins identified in the Batelli gland of *Mahanarva spectabilis* nymphs. (a) Distribution of the 20 most frequent Pfam domains, highlighting conserved structural motifs and enzymatic modules prevalent in the dataset. (b) Gene Ontology (GO) annotation of the 20 most enriched terms, including categories related to molecular function, biological process and cellular component, illustrating the functional diversity of the glandular proteome. (c) Top 20 KEGG pathways represented among the identified proteins, revealing metabolic and signalling routes potentially associated with secretion, foam stabilization and insect‐plant interactions. (d) Top 20 KOG functional categories, organized by higher‐order cellular and metabolic processes, reflecting the evolutionary and physiological context of protein functions in the Batelli gland.

Proteomic analysis of the Batelli gland secretome identified a diverse set of 500 proteins spanning functional categories associated with cellular homeostasis, stress adaptation and interactions with the host environment (Figure 5 and Table S9). Functional annotation of the Batelli gland proteome using PFAM, KEGG and Gene Ontology (GO) revealed a broad representation of domains and pathways related to stress tolerance, metabolic activity and extracellular secretion.

The PFAM domain analysis highlighted abundant protein families, such as glutathione S‐transferases (23 hits), EF‐hand calcium‐binding proteins (19 hits) and chaperone‐related domains, consistent with roles in detoxification, signalling and protein folding. A substantial proportion of proteins carried general N‐ and C‐terminal domains, suggesting conserved structural motifs, while others were linked to enzymatic functions including oxidoreductases and transferases.

The KEGG classification retrieved 103 proteins without a defined annotation, but among the functionally characterized entries, enriched categories included mitochondrial proteins (29 hits), enzymes of detoxification pathways and transport‐related functions. Several entries mapped to metabolic routes relevant for host adaptation, including xenobiotic metabolism and energy production.

The GO annotation further supported these findings. The most represented terms included ATP binding (61 proteins), reflecting high energy demand; cytoplasmic localization (41 proteins) and membrane‐associated functions (37 proteins), many of which corresponded to ion transporters and enzymes. Biological process terms highlighted stress responses and protein metabolic processes, aligning with the predicted roles of the gland in maintaining protein stability and contributing to foam formation. Notably, 35 proteins (≈7% of the dataset) remained without functional annotation, highlighting the presence of potentially novel or poorly characterized components of the gland proteome.

Protein identification performed using the PEAKS algorithm from LC–MS/MS data allowed the inference of relative protein abundance in the samples based on the spectral counting approach. Thus, proteins with a higher number of unique peptides and/or greater sequence coverage were considered to be relatively more abundant, as these parameters are commonly associated with higher protein abundance in label‐free proteomic analyses. These results provide insight into the predominant protein constituents of each foam‐associated band and serve as the basis for subsequent functional and structural annotation.

When protein abundance was inferred from the number of detected peptides, the dataset was dominated by highly expressed chaperones, posttranslational regulators and metabolic enzymes (Table 4). The top‐ranking proteins included multiple HSP70 homologues (e.g., DnaK‐like proteins) with over 80 peptides, emphasizing their critical role in proteostasis. Other abundant chaperones included 60 kDa chaperonins, each represented by nearly 70 peptides, highlighting the gland's strong investment in protein folding and stabilization.

**TABLE 4:** Top 15 most abundant Batelli gland proteins from proteomic analysis.

Protein ID	Peptide count	Functional category	Annotation/matched term
Gene 28070	81	chaperones	Chaperone protein DnaK [Stylophora pistillata]
Gene 109130	79	Posttranslational modification	protein turnover
Gene 28076	79	Gene 28,076.g.28076.m.28076	Osmoregulation transport
Gene 26924	72	Gene 26924	Gene 26924
Gene 29120	69	chaperones	60 kDa chaperonin [Stylophora pistillata]
Gene 86297	69	Gene 86,297.g.86297	Gene 86297.g.86297.m.86297
Gene 29122	69	chaperones	60 kDa chaperonin [Stylophora pistillata]
Gene 25378	67	protein turnover	chaperones
Gene 26709	64	m.26709	Gene 26,709.g.26709
Gene 73327	63	m.73327	Gene 73,327.g.73327
Gene 28072	60	hypothetical protein B566_EDAN000458	Gene 28072
Gene 38,689	60	Gene 38,689.g.38689.m.38689	Cuticle barrier
Gene 25201	58	Unknown	Unknown
Gene 28090	58	ATP synthase subunit beta	mitochondrial [Nilaparvata lugens]
Gene 3153	58	m.3153	Gene 3153.g.3153

Proteins associated with posttranslational modification and protein turnover were also highly represented (>70 peptides), underscoring intense regulation of protein quality control. Candidates linked to osmoregulation and membrane transport were detected at similar levels, suggesting active ion homeostasis and fluid balance within the gland. Metabolic resilience was reflected in the detection of an ATP synthase β‐subunit with nearly 60 peptides, pointing to sustained energy demands. Structural adaptation was further suggested by proteins annotated under cuticle barrier remodelling, while several abundant yet uncharacterized proteins (60–70 peptides) may represent novel contributors to gland physiology. This proteomic profile underscores the Batelli gland's emphasis on protein stability, metabolic activity, osmoregulation and extracellular barrier functions. These features are consistent with its specialized role in secreting protective foam enriched in structural and defensive proteins.

### Candidate proteins potentially relevant to host–plant interaction and environmental survival from proteomic analysis

**FIGURE 6:**
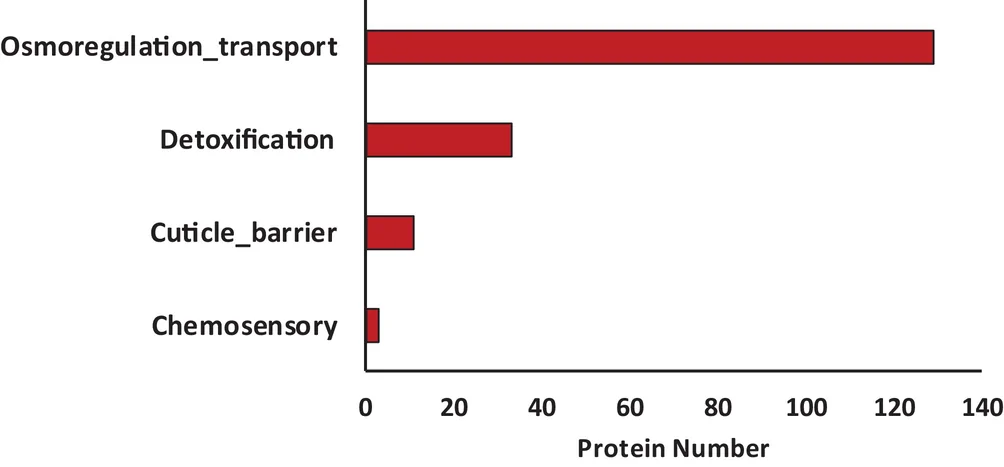
Distribution of proteins by functional category from proteomic analysis of Batelli gland. Bars show the number of proteins annotated per category (Detoxification, Chemosensory, Cuticle/Barrier, Osmoregulation/Transport).

Proteomic analysis of the Batelli gland identified several protein groups potentially associated with host–plant interaction and environmental adaptation (Figure 6 and Table S10). Among the annotated proteins, 33 candidates were assigned to detoxification‐related functions, including cytochrome P450 monooxygenases, glutathione S‐transferases (GSTs) and other oxidoreductases. These proteins are commonly linked to the processing of plant‐derived compounds and xenobiotics and were well represented in the gland proteome.

A smaller set of three proteins was classified as chemosensory‐related, comprising odorant‐binding proteins (OBPs/PBPs) and chemosensory proteins (CSPs). Their presence in the Batelli gland proteome suggests a potential association with the perception or handling of chemical cues at the interface between the insect, the foam and the host plant.

Proteins involved in osmoregulation and ion transport constituted a prominent fraction of the dataset, with 129 annotated proteins, including V‐ATPase subunits, Na^+^/K^+^‐ATPases, solute carrier transporters and carbonic anhydrases. These proteins are typically associated with ionic and osmotic homeostasis and may reflect physiological requirements linked to feeding on xylem sap. In addition, 11 proteins were assigned to cuticle and barrier remodelling, including structural cuticular proteins and enzymes related to chitin metabolism.

## DISCUSSION

The spittlebug *M. spectabilis* is an insect pest of forage grasses, as both adults and nymphs feed on xylem sap, causing substantial reductions in plant regrowth, quality and productivity. Nymphs are particularly damaging because they remain attached to basal plant tissues for extended periods, produce protective foam and create conditions that predispose hosts to secondary infections, further accelerating pasture decline. As such, strategies to combat spittlebug outbreaks in a sustainable way are being researched. These include the induction of plant resistance to spittlebug attacks (Alvarenga et al., 2017, 2019; Leite et al., 2014), diversification of pastures (Alvarenga et al., 2017), the use of plant compounds as control agents (Dias et al., 2019; Nascimento et al., 2021), soil fertilization (Aguiar et al., 2014; Alvarenga et al., 2019) and the use of entomopathogenic fungi (Campagnani et al., 2017; Campagnani et al., 2024). Added to these strategies, the molecular adaptations that sustain their survival on nutrient‐poor xylem sap and their ability to resist plant defences have been proposed in this current research. Nymphs of *M. spectabilis* also stand out because they produce a characteristic foam that encases their bodies.

This foam, produced by secretions of the Batelli gland in combination with excretory fluids associated with the Malpighian tubules, has been described in spittlebugs as a multifunctional barrier against desiccation, temperature fluctuations, predation and microbial growth (Rakitov, 2002; Szterk et al., 2024; Tonelli et al., 2020).

Recent studies on other spittlebug species, such as *M. fimbriolata* and *Poophilus costalis*, confirm that these foams contain complex mixtures of proteins, carbohydrates and lipids that ensure thermal stability and antimicrobial protection (Sahayaraj et al., 2021; Tonelli et al., 2018). By focusing on the transcriptome of nymphs and the proteome of the Batelli gland, our work offers a comprehensive view of the molecular components underlying foam production and their potential functions in plant–insect–environment interactions.

Assembly statistics confirmed the reliability of the dataset, with an N50 length in the expected range for insect transcriptomes and sufficient representation across different length categories. The presence of both short and long unigenes indicates efficient assembly across highly and moderately expressed genes, providing a valuable resource for gene discovery and expression analysis.

RNA‐seq of nymphs provided a genome‐wide resource of expressed genes, annotated across Pfam, KEGG and GO categories, while LC–MS/MS proteomics revealed hundreds of proteins secreted by the Batelli gland. The integration of these datasets enabled the identification of genes and proteins with predicted functions in detoxification, chemosensory perception, osmoregulation, stress responses, immunity and cuticle remodelling. Comparable integrative approaches have been successfully applied in other hemipterans, such as the leafhopper *Nephotettix cincticeps* (Wu et al., 2023) and the aphid *Metopolophium dirhodum* (Gebrekidan et al., 2024), confirming that transcriptome‐proteome coupling is a powerful strategy to resolve the molecular basis of secreted structures in sap‐feeding insects.

We detected a large repertoire of detoxification genes, including CYPs, GSTs, UGTs, CCEs and AKRs. These gene families were strongly enriched in KEGG pathways such as glutathione metabolism and drug/xenobiotic metabolism. Detoxification enzymes are widely recognized as central mediators of host adaptation in hemipterans, as shown for *Empoasca vitis* nymphs, where many detoxification genes are upregulated during feeding (Shao et al., 2017). Similarly, expansions of CCEs, GSTs and P450s were reported in *Picromerus lewisi* (Li et al., 2022), highlighting their evolutionary importance in insect‐plant interactions. Notably, glutathione metabolism has also been reported as a key pathway associated with resistance in *Paspalum regnellii* under *M. spectabilis* attack, highlighting its central role in modulating both plant defence responses and insect detoxification processes within this interaction (Begnami et al., 2025). The robust detoxification repertoire in *M. spectabilis* likely reflects adaptation to allelochemicals and secondary metabolites from forage grasses.

Chemosensory transcripts identified here include odorant‐binding proteins (OBPs), pheromone‐binding proteins (PBPs), chemosensory proteins (CSPs), and both gustatory and olfactory receptors. Such proteins play essential roles in detecting host‐derived volatiles and are crucial for orientation, feeding and oviposition. In aphids such as *Myzus persicae*, RNA‐seq analyses have shown that chemosensory gene expression varies between nymphs and adults, reflecting developmental and ecological needs (Sharma et al., 2024). The high expression of PBP/GOBP family members in *M. spectabilis* may similarly be related to host localization and gregarious nymphal behaviour within foams.

Feeding on xylem sap presents unique osmotic challenges due to high water content and low nutritional value. Our data revealed nearly 4000 transport‐related transcripts, including multiple V‐ATPase subunits, Na^+^/K^+^‐ATPases, aquaporins, solute carriers and ion transporters. Carbonic anhydrases and proton‐pumping ATPases were among the most abundant. These findings align with observations in cicadas and other xylem feeders, where osmoregulatory machinery is highly expanded (Beckett et al., 2019). Such adaptations enable insects to maintain ionic and osmotic balance while processing massive volumes of dilute sap.

We identified transcripts encoding cuticular proteins (CPRs) and chitin metabolic enzymes, which are primarily associated with exoskeleton remodelling. Their presence among Batelli gland‐associated transcripts suggests a potential, indirect contribution to the foam environment, possibly by reinforcing the interface between the insect body and the foam or by providing structural or antimicrobial properties. This interpretation is supported by reports in *Aphrophora alni*, where cuticle‐ and chitin‐associated components were detected in spittlebug foam (Szterk et al., 2024). Nevertheless, the role of these proteins in foam stability should be regarded as a functional hypothesis rather than a demonstrated mechanism. The identified transcripts for CPRs and chitin metabolic enzymes suggest remodelling of the exoskeleton and contributions to foam structure. This is consistent with reports in *Aphrophora alni*, where proteins and chitin‐associated components were detected in foam (Szterk et al., 2024). The cuticle and foam thus appear to form an integrated barrier that protects nymphs against mechanical damage and microbial invasion.

Abundant transcripts for heat shock proteins and antioxidant enzymes confirm the importance of stress tolerance. In addition, immunity‐related transcripts such as serine proteases, serpins, lysozymes and peptidoglycan recognition proteins (PGRPs) reflect the need for defence within foam habitats (Lu et al., 2014). Antimicrobial activity has been demonstrated in spittlebug foams (Tonelli et al., 2018), and predicted lysozyme‐like proteins were also predicted in our secretome, strengthening the link between secreted proteins and microbial defence.

Approximately 16% of expressed loci were classified as putative alternatively spliced, including detoxification enzymes, receptors and immune‐related genes. This level of alternative splicing is comparable to that reported for other hemipteran and insect transcriptomes assembled de novo, where 10%–30% of genes commonly exhibit multiple isoforms (Begnami et al., 2026; Graveley et al., 2011), indicating a moderate and computationally inferred level of isoform diversity. Such transcriptomic plasticity is consistent with findings in aphids and leafhoppers, where host plant shifts drive rapid gene expression adjustments (Chongtham et al., 2024; Rivera‐Vega et al., 2017). Alternative splicing may thus be a crucial mechanism enabling *M. spectabilis* to adapt to diverse pasture hosts and environmental stressors.

SignalP and Phobius analyses predicted 168 predicted candidate secreted proteins in the *M. spectabilis*, including multiple detoxification enzymes, structural proteins, various proteases, immune‐related factors, chaperones and storage/transport proteins. Function prediction was supported by annotation in Pfam, KEGG and GO databases, and protein identity was validated through LC/MS evidence. Similar integrative proteome‐transcriptome approaches in sap‐feeding Hemiptera, such as aphids and leafhoppers, have revealed sets of secreted proteins implicated in plant–insect interaction, detoxification and defence modulation (Wu et al., 2023; Zhang et al., 2023). These studies demonstrate that multi‐omics analyses can systematically identify and functionally characterize the proteins contributing to successful sap‐feeding and adaptation to diverse host plants.

We detected high levels of detoxification enzymes and transporters in the Batelli gland proteome, indicating a secretory role in the processing of host‐derived xenobiotics. Proteases and protease inhibitors were strongly represented, suggesting both remodelling of the foam matrix and the activation of immune cascades, as has been observed in other hemipteran salivary proteomes (da Silva Vaz Junior et al., 2024). Structural proteins, such as cuticle proteins, and chitin metabolic enzymes were also present, supporting their contribution to foam stability, a feature demonstrated in biofoam analyses of spittlebugs (Sahayaraj et al., 2021). Immune proteins, including lysozymes and phenoloxidases, as well as molecular chaperones and folding factors like BiP and protein disulfide isomerases (PDIs), reinforce the multifunctional nature of the foam matrix (Hendershot et al., 2024). Notably, the identification of storage proteins, including hexamerins and lipocalins, emphasizes additional buffering and carrier roles for secreted proteins.

The Batelli gland proteome demonstrates the multifunctionality and ecological importance of spittlebug foam. It serves as a biochemical and structural barrier, ensures osmotic regulation, mediates detoxification of plant allelochemicals and provides antimicrobial defence. Ecological studies have confirmed that spittlebug foam maintains a favourable microclimate and shields against desiccation and predators (Beckett et al., 2019; Tonelli et al., 2018). The large fraction of unannotated secreted proteins suggests the presence of novel, possibly spittlebug‐specific components, which may represent unique evolutionary innovations contributing to the adaptive success of *M. spectabilis*.

Chemically, spittlebug foam is a complex, amphipathic and viscoelastic material containing proteins (including glycopeptides and proteoglycans), carbohydrates and, depending on the species, fatty acids such as palmitic and stearic acids. This mixture is consistent with a structural matrix capable of capturing air and resisting collapse. Functionally, the froth buffers temperature and humidity, shields from light and impedes natural enemies, while antimicrobial properties, including antibacterial and antifungal activities, have also been reported. Microbial communities have been described in the foams of related *Mahanarva* species, suggesting additional ecological interactions modulated by foam chemistry (Sahayaraj et al., 2021).

Consistent with these reports, the Batelli gland‐derived, SignalP‐positive proteins characterized in this study likely constitute the proteinaceous core of the foam and support multiple, non‐exclusive functions. Secreted, glycosylated and low‐complexity proteins, such as mucin‐like and Pro‐/Thr‐/Ser‐rich domains, together with proteoglycan‐associated modules, may contribute to viscosity, reduce surface tension and enhance bubble persistence, in line with historic descriptions of glycopeptides and proteoglycans in spittle. In parallel, secreted hydrolases and redox enzymes, including candidate chitinases and oxidoreductase‐like proteins, may underpin the reported antifungal and antibacterial activities of the foam, contributing to the control of microbial colonization in the nymphal microhabitat. Other proteins with surfactant‐like properties, together with lipid‐binding or lipid‐transfer proteins, are compatible with the fatty acids documented in spittle and could maintain a stable bubble film that regulates temperature, light exposure and rheological properties. Mechanistically, the foam is generated by periodic abdominal pumping that injects air into the secretions of the Batelli glands and Malpighian tubules. The Batelli gland output specifically facilitates bubble formation, rendering the anal fluid spumous, as described in classical and modern accounts. Thus, the most coherent interpretation is that the Batelli gland is the primary source of the protein and lipid components that, together with polysaccharides, build the extracellular protective foam matrix, and the SignalP‐positive proteins identified here represent immediate candidates secreted to form and maintain this biofoam.

Overall, the Batelli gland secretome aligns with the established view that these glandular secretions are both necessary and sufficient to produce the spittlebug foam. By providing a proteinaceous scaffold enriched in glycoproteins, surfactants and antimicrobial enzymes, in combination with lipids and carbohydrates, the Batelli gland ensures bubble stability, microclimate regulation and a first defensive barrier during the xylem‐feeding nymphal stage of *M. spectabilis*.

## CONCLUDING REMARKS

By integrating transcriptomic and proteomic data, this study delivers the first in‐depth molecular characterization of the nymphal stage in *M. spectabilis*. The identification of multiple functional categories, including detoxification enzymes (e.g., cytochrome P450s, GSTs), chemosensory receptors (OBPs, CSPs, PBPs), osmoregulatory proteins (aquaporins, ATPases), cuticular proteins and chitin metabolic enzymes, stress response factors (heat shock proteins, antioxidant enzymes) and diverse secreted proteins—highlights the multifactorial adaptations that empower spittlebug nymphs to survive and develop in the chemically and physically stressful environment of forage grasses.

These results corroborate and extend findings from recent transcriptomic and proteomic analyses of other hemipterans, advancing our understanding of the molecular and ecological strategies underlying sap‐feeding insect adaptation. Importantly, this molecular framework may serve as a foundation for the development of innovative, targeted management strategies for spittlebug pests in pasture ecosystems, including approaches based on disruption of key protein families or foam‐related physiological processes.

## AUTHOR CONTRIBUTIONS

**Monique da Silva Bonjour**: Methodology; formal analysis; writing—review and editing; writing—original draft; software; investigation. **Angelo José Rinaldi**: Formal analysis; writing—original draft; writing—review and editing; methodology; software. **Eulálio Gutemberg Dias dos Santos**: Formal analysis; methodology; software. **Gabriely Teixeira Bhering Faria**: Formal analysis; methodology. **Ana Márcia Escocard de Azevedo Manhães**: Formal analysis; methodology. **Jorge Fernando Pereira**: Methodology; conceptualization; writing—original draft; writing—review and editing; project administration; funding acquisition; investigation. **Alexander Machado Auad**: Conceptualization; writing—original draft; writing—review and editing; project administration; methodology; funding acquisition; investigation. **Maria Goreti Almeida Oliveira**: Conceptualization; methodology; writing—review and editing; writing—original draft; project administration; supervision; funding acquisition; investigation. **Humberto Josué de Oliveira Ramos**: Conceptualization; formal analysis; data curation; writing—original draft; writing—review and editing; supervision; methodology; software; funding acquisition; project administration.

## CONFLICT OF INTEREST STATEMENT

All authors have no pecuniary or other personal interest.

## ETHICS STATEMENT

The work did not involve ethical questions, and all authors agreed to participate in this research study.

## CONSENT FOR PUBLICATION

All authors are in accordance with the publication of the manuscript.

## Supporting information


**Figure S1.** Average error rate along Illumina paired‐end reads of *Mahanarva spectabilis* nymph RNA‐seq. The error rate remained extremely low (<0.0003) across Read1 and Read2, with only a slight increase toward the end of the reads, consistent with typical Illumina sequencing profiles.

**Figure S2.** Base composition along Illumina paired‐end reads of *Mahanarva spectabilis* nymph RNA‐seq. After the first ~20 cycles, nucleotide proportions stabilized at ~30% for A and T and ~20% for G and C, indicating balanced base distribution. The proportion of undetermined bases (N) was negligible across both reads.

**Figure S3.** Length distribution of assembled unigenes from the *Mahanarva spectabilis* nymph transcriptome. Most unigenes ranged between 300 and 1000 nucleotides, with 8.6% exceeding 2000 nt, representing putative full‐length transcripts.

**Figure S4:** (A) Random distribution of reads along transcripts in the *Mahanarva spectabilis* nymph RNA‐seq library. (B) Reads were evenly distributed across the 5′–3′ gene regions, with only minor central enrichment. (C) Insert size distribution of paired‐end reads, showing a peak at ~400 bp. 4D Gene saturation curve (FPKM ≥0.1) indicating that sequencing depth was sufficient to recover nearly all expressed transcripts.

**Table S1.** Comprehensive functional annotation of transcripts expressed in *Mahanarva spectabilis* nymphs based on RNA‐Seq data. Each entry corresponds to a predicted protein‐coding transcript with its respective gene equivalence ID and functional annotations derived from multiple databases, including Pfam (protein domains), Gene Ontology (molecular function and biological process), KEGG (metabolic pathways), and other functional classifications. This dataset provides the reference framework used to identify putative secreted and functionally relevant proteins expressed in the Batelli gland.

**Table S2.** Functional annotation of *Mahanarva spectabilis* nymph transcripts related to plant‐insect and environmental interactions. The table lists annotated genes grouped by major biological categories, including signal transduction, stress and defence responses, digestion, metabolism, and environmental adaptation. Functional classification was based on Gene Ontology (GO) terms, KEGG pathways, and Pfam domains inferred from RNA‐Seq transcript annotations.

**Table S3.** Summary of gene counts by functional category identified in the *Mahanarva spectabilis* nymph transcriptome. Categories represent major functional groups inferred from RNA‐Seq annotations, including detoxification, chemosensory perception, cuticle and barrier formation, and osmoregulation and transport processes. The number of unique genes assigned to each category is indicated.

**Table S4.** Distribution of transcript isoforms per gene in the *Mahanarva spectabilis* nymph transcriptome assembly. The table shows the number of genes producing a given number of transcript isoforms, ranging from single‐isoform genes to highly alternatively spliced loci generating more than 40 isoforms. This distribution highlights the prevalence of genes with one or two isoforms and the relatively small number of loci exhibiting extensive alternative splicing.

**Table S5.** Summary of alternatively spliced genes in the *Mahanarva spectabilis* nymph transcriptome. The table lists genes exhibiting multiple transcript isoforms (≥2) grouped by major functional categories, including metabolism, signal transduction, stress response, and structural components. These data provide an overview of the functional distribution of alternatively spliced loci identified from RNA‐Seq analysis.

**Table S6.** Functional classification of alternatively spliced genes associated with plant‐environment interactions in the *Mahanarva spectabilis* nymph transcriptome. The table lists genes exhibiting two or more transcript isoforms, grouped by functional categories related to plant responses, environmental adaptation, and stress signalling, as inferred from RNA‐Seq‐based splicing analysis.

**Table S7.** Prediction of secreted proteins (secretome) in the *Mahanarva spectabilis* nymph transcriptome. The table lists protein‐coding transcripts predicted to contain signal peptides or secretion‐associated features identified using SignalP and Phobius. These results were derived from RNA‐Seq data and represent putative secreted proteins potentially involved in extracellular or glandular functions.

**Table S8.** Functional annotation of predicted secreted proteins (secretome) from the *Mahanarva spectabilis* nymph transcriptome. The table lists putative secreted proteins identified through SignalP and Phobius prediction, along with their associated Gene Ontology (GO) terms, KEGG pathways, and Pfam domains. This functional analysis provides insight into the potential biological roles of the secreted proteins expressed in the Batelli gland.

**Table S9.** Functional annotation of proteins identified in the Batelli gland of *Mahanarva spectabilis* nymphs by LC–MS/MS proteomic analysis. The table lists experimentally detected proteins along with their functional classifications, including Pfam domains, Gene Ontology (GO) terms, KEGG pathways, and categories related to metabolism, stress response, and secretion. These annotations provide insights into the molecular composition and potential biological roles of the Batelli gland secretome.

**Table S10.** Functional annotation of Batelli gland proteins from *Mahanarva spectabilis* nymphs related to plant‐host and environmental interactions. Proteins were identified by LC–MS/MS and annotated according to Gene Ontology (GO) terms, KEGG pathways, and Pfam domains. The dataset highlights proteins potentially involved in host recognition, defence modulation, stress adaptation, and other processes relevant to plant.

## Data Availability

All scripts and datasets underpinning the analyses, tables and figures are publicly available at Bonjour et al. (2026), Zenodo: https://doi.org/10.5281/zenodo.18681941. Raw sequencing data are deposited in the NCBI Sequence Read (Ramos et al., 2026b), archive under BioProject PRJNA1404840 (https://www.ncbi.nlm.nih.gov/bioproject/?term=PRJNA1404840). The mass spectrometry proteomics data have been deposited to the MassIVE repository and are publicly available via the ProteoSAFe platform under accession number MSV000101533 (Ramos et al., 2026a). The dataset is also accessible through the https://doi.org/10.25345/C59Z90S0P.
