# Supplementary material for: Transcriptomic and proteomic signatures underlying nymphal adaptation and foam production in the forage pest M ahanarva spectabilis
Source: Insect Mol Biol. 2026 Jun 12;35(5):556–75. doi: 10.1111/imb.70050 (PMC13551515; doi:10.1111/imb.70050)
Supplement: Supplementary file 1 [file IMB-35-556-s003.docx]

**Table S3.** Summary of gene counts by functional category identified in the Mahanarva spectabilis nymph transcriptome. Categories represent major functional groups inferred from RNA-Seq annotations, including detoxification, chemosensory perception, cuticle and barrier formation, and osmoregulation and transport processes. The number of unique genes assigned to each category is indicated.

| **Category** | **Unique_Genes** |
| --- | --- |
| Detoxification | 797 |
| Chemosensory | 53 |
| Cuticle_barrier | 158 |
| Osmoregulation_transport | 3965 |
