# Supplementary material for: Transcriptomic and proteomic signatures underlying nymphal adaptation and foam production in the forage pest M ahanarva spectabilis
Source: Insect Mol Biol. 2026 Jun 12;35(5):556–75. doi: 10.1111/imb.70050 (PMC13551515; doi:10.1111/imb.70050)
Supplement: Supplementary file 3 [file IMB-35-556-s007.docx]

**Table S4.** Distribution of transcript isoforms per gene in the Mahanarva spectabilis nymph transcriptome assembly. The table shows the number of genes producing a given number of transcript isoforms, ranging from single-isoform genes to highly alternatively spliced loci generating more than 40 isoforms. This distribution highlights the prevalence of genes with one or two isoforms and the relatively small number of loci exhibiting extensive alternative splicing.

| **Isoforms**  **per_gene** | **Gene Numbers** |
| --- | --- |
| 1 | 83295 |
| 2 | 7881 |
| 3 | 3420 |
| 4 | 1881 |
| 5 | 1085 |
| 6 | 700 |
| 7 | 455 |
| 8 | 287 |
| 9 | 216 |
| 10 | 141 |
| 11 | 88 |
| 12 | 56 |
| 13 | 36 |
| 14 | 38 |
| 15 | 25 |
| 16 | 13 |
| 17 | 10 |
| 18 | 10 |
| 19 | 6 |
| 20 | 5 |
| 21 | 4 |
| 22 | 4 |
| 23 | 1 |
| 24 | 3 |
| 25 | 1 |
| 27 | 1 |
| 28 | 1 |
| 30 | 1 |
| 38 | 1 |
| 39 | 1 |
| 43 | 1 |
| 44 | 1 |
| 75 | 1 |
