# Supplementary material for: Transcriptomic and proteomic signatures underlying nymphal adaptation and foam production in the forage pest M ahanarva spectabilis
Source: Insect Mol Biol. 2026 Jun 12;35(5):556–75. doi: 10.1111/imb.70050 (PMC13551515; doi:10.1111/imb.70050)
Supplement: Supplementary file 5 [file IMB-35-556-s009.docx]

**Figure S3.**
